# Liver enzymes and metabolic syndrome: a large-scale case-control study

**DOI:** 10.18632/oncotarget.5792

**Published:** 2015-09-22

**Authors:** Lu Zhang, Xiangyu Ma, Zhi Jiang, Kejun Zhang, Mengxuan Zhang, Yafei Li, Xiaolan Zhao, Hongyan Xiong

**Affiliations:** ^1^ Department of Epidemiology, College of Preventive Medicine, Third Military Medical University, Chongqing, China; ^2^ Division of Scientific Research, Third Military Medical University, Chongqing, China; ^3^ Health Care Center of Southwest Hospital, The First Affiliated Hospital of The Third Military Medical University, Chongqing, China; ^4^ Department of Clinical Laboratory, Institute of Surgery Research, Daping Hospital, Third Military Medical University, Chongqing, China

**Keywords:** metabolic syndrome, liver enzymes, association, biomarker, Pathology section

## Abstract

Previous studies suggested that elevated liver enzymes could be used as potential novel biomarkers of Metabolic syndrome (MetS) and its clinical outcomes, although the results were inconsistent and the conclusions were underpowered. A case-control study with 6,268 MetS subjects and 6,330 frequency-matched healthy controls was conducted to systematically evaluated levels of four liver enzymes (ALT, AST, GGT and ALP), both in overall populations and in subjects with normal liver enzymes, with MetS risk using both quartiles and continuous unit of liver enzymes. We found significant associations were detected for all above analyses. Compared with quartile 1 (Q1), other quartiles have significant higher MetS risk, with ORs ranging from 1.15 to 18.15. The highest effected was detected for GGT, for which the OR value for the highest versus lowest quartile was 18.15 (95% CI: 15.7-20.9). Mutual adjustment proved the independence of the relations for all four liver enzymes. Sensitivity analyses didn’t materially changed the trend. To the best of our knowledge, this study should be the largest, which aimed at evaluating the association between liver enzymes measures and MetS risk. The results can better support that liver enzyme levels could be used as clinical predictors of MetS.

## INTRODUCTION

Metabolic syndrome (MetS) is a constellation of conditions, increased blood pressure, high blood sugar level, excess body fat around the waist and abnormal cholesterol levels, that occur together, increasing the risk of heart disease, stroke, insulin resistance, diabetes, and nonalcoholic fatty liver disease (NAFLD) [1–4]. According to the Third National Health and Nutrition Examination Survey of United States, the prevalence of MetS has been an estimated 34% of the adult population [5]. The predominant underlying risk factor for the s MetS appear to be abdominal obesity [6–8]. However, not all obese develop the syndrome and even lean individuals can be insulin resistant, which was highly related with MetS [9]. The MetS can be clinically manifested in a variety of ways, which brings some difficulties for the clinical diagnosis of MetS. Recent experimental and clinical studies showed that liver enzymes might be novel candidate biomarkers for MetS and its clinical outcomes [10–13].

To present, plasma level of liver enzymes, including Alanine transaminase (ALT) and Aspartate aminotransferase (AST), commonly used as the indicators of liver damage, and Gamma-Glutamyl Transpeptidase (GGT), a biomarker for oxidative stress associated with glutathione regulation, and alkaline phosphatase (ALP), were widely explored as the indicators of MetS and its components among different populations [14–25]. However, the results were inconsistent, and sample size were limited and underpowered (most of the sample size was smaller than 1000). Given the plausible role for liver enzymes in MetS development and the conflicting results of previous studies, we conducted this study to strength the understanding of relations between liver enzymes and MetS risk, using: (1) a frequency matched case-control study design; (2) a large sample size among Chinese adolescents (6,268 MetS subjects and 6,330 healthy controls); (3) we performed the studies in the overall sample and in participants with normal liver enzyme values.

## RESULTS

Totally included in this study were 6,268 MetS subjects and 6,330 healthy controls. As shown in Table 1, MetS subjects are slightly elder than healthy controls, lower educated, more like to be smokers and drinkers. MetS subjects also have lower participating rate of sporting, compared with healthy controls. Table 1 also lists the comparison of five MetS components and four liver enzymes levels between MetS subjects and healthy controls. Obviously, the values in MetS subjects were higher than those in healthy controls.

**Table 1:** Clinical characteristics of the study population

	MetS subjects	Healthy controls	P value ^a^
Participants (N)	6,268	6,330	
Age (Years)	44.9 ± 10.4	42.1 ± 8.7	P < 0.001
Gender (%)			
Male	78.7	77.4	0.084
Female	21.3	22.6	
Education (%)			
College and above	75.1	79.4	P < 0.001
Lower than college	24.9	20.6	
Smoking status			
Never	49.8	54.7	P < 0.001
Ever	6.4	5.0	
current	43.8	40.3	
Drinking			
Never	21.8	28.3	P < 0.001
Ever	10.2	9.8	
current	68.0	61.9	
Sporting			
Yes	56.8	60.2	0.001
No	43.2	39.8	
Waist circumference	90.4 ± 8.1	77.9 ± 6.1	P < 0.001
Triglycerides	3.5 ± 2.8	1.1 ± 0.3	P < 0.001
HDL cholesterol	1.2 ± 0.3	1.6 ± 0.3	P < 0.001
Blood pressure			
systolic pressure	138.2 ± 17.5	112.1 ± 9.3	P < 0.001
diastolic pressure	88.5 ± 12.2	70.9 ± 7.7	P < 0.001
Fasting glucose	6.4 ± 1.9	5.1 ± 0.3	P < 0.001
ALT	31.7 ± 13.4	22.8 ± 10.7	P < 0.001
AST	30.3 ± 10.3	26.7 ± 7.3	P < 0.001
GGT	52.8 ± 50.5	25.3 ± 21.0	P < 0.001
ALP	89.6 ± 24.1	80.7 ± 22.3	P < 0.001

MetS: metabolic syndrome; BMI: body mass index; ALT: Alanine transaminase; AST: Aspartate aminotransferase; GGT: Gamma-Glutamyl Transpeptidase; ALP: alkaline phosphatase.

a Continuous variables: mean values ± standard deviation, *p*-value from *t*-tests; Categorical variables: percentages, *p*-values from x^2^ test.

Table 2 shows the results of logistic regression analysis for the presence of MetS in relation with liver enzymes adjusting for age, gender, and education level. We first explored the associations between MetS risk and quartiles of liver enzymes. We set the first quartile as the referent, and all of the four liver enzymes showed statistically significant linear increased risk (*P* < 0.001). Compared with quartile 1 (Q1), other quartiles have significant higher MetS risk, with ORs ranging from 1.15 to 18.15. The highest effected was detected for GGT, for which the OR value for the highest versus lowest quartile was 18.15 (95% CI: 15.7-20.9). Furthermore, We also analyzed the associations between MetS risk and continuous unit of liver enzymes. Significant trends were found for increasing unit of ALT, AST, GGT and ALP (per 5 unit). The smallest effect size was for ALP (OR: 1.09, 95% CI: 1.08-1.10), and the largest was for ALT (OR: 1.41, 95% CI: 1.38-1.43). We then conducted stratified analyses by gender for the relations between MetS risk and liver enzymes, using both quartiles and continuous unit of liver enzymes (Table 3). The ORs was bigger in males for ALT and AST, while the ORs was bigger in females for GGT and ALP. In mutually adjusted models which include all of the four liver enzymes in the logistic regression model, they all remained significantly associated with MetS risk, which means the independence of their relations.

**Table 2:** Logistic regression analysis for the presence of MetS in relation with liver enzymes

	Overall				
	MetS subjects	Healthy controls	OR^a^	95% CI	P value
**ALT**					
Q1 (<15)	455	1,398	referent		
Q2 (15–20)	823	1,697	1.50	1.31–1.72	
Q3 (20–27)	1,352	1,520	3.00	2.63–3.43	
Q4 (>27)	3,638	1,715	8.03	7.06–9.12	
Continuous (Per 5 unit increase)			1.41	1.38–1.43	P < 0.001
**AST**					
Q1 (<22)	782	1,423	referent		
Q2 (22–25)	873	1,352	1.15	1.02–1.31	
Q3 (25–30)	1,774	1,917	1.63	1.46–1.82	
Q4 (>30)	2,839	1,638	3.06	2.75–3.41	
Continuous (Per 5 unit)			1.33	1.29–1.36	P < 0.001
**GGT**					
Q1 (<16)	376	1,701	referent		
Q2 (16–20)	495	1,398	1.90	1.62–2.22	
Q3 (20–28)	1,151	1,627	4.32	3.74–5.00	
Q4 (>28)	4,246	1,604	18.15	15.7–20.9	
Continuous (Per 5 unit)			1.27	1.25–1.29	P < 0.001
**ALP**					
Q1 (<66)	920	1,674	referent		
Q2 (66–78)	1,177	1,489	1.43	1.28–1.60	
Q3 (78–93)	1,692	1,622	1.87	1.68–2.08	
Q4 (>93)	2,479	1,545	2.80	2.52–3.10	
Continuous (Per 5 unit)			1.09	1.08–1.10	P < 0.001

a adjusted for age, gender, and education level

**Table 3:** Logistic regression analysis for the presence of MetS in relation with liver enzymes stratified by gender

	Males			Females		
	OR^b^	95% CI	P value	OR^b^	95% CI	P value
**ALT**						
Q1 (<15)	referent			referent		
Q2 (15–20)	1.71	1.42–2.07		1.37	1.11–1.67	
Q3 (20–27)	3.64	3.05–4.34		2.43	1.94–3.05	
Q4 (>27)	10.1	8.57–12.0		4.77	3.79–6.01	
Continuous (Per 5 unit increase)	1.43	1.39–1.45	P < 0.001	1.33	1.28–1.39	P < 0.001
**AST**						
Q1 (<22)	referent			referent		
Q2 (22–25)	1.35	1.17–1.57		0.88	0.70–1.09	
Q3 (25–30)	1.90	1.67–2.18		1.28	1.04–1.57	
Q4 (>30)	3.80	3.33–4.32		1.76	1.42–2.17	
Continuous (Per 5 unit)	1.37	1.33–1.41	P < 0.001	1.18	1.12–1.25	P < 0.001
**GGT**						
Q1 (<16)	referent			referent		
Q2 (16–20)	1.40	1.10–1.77		2.81	2.27–3.50	
Q3 (20–28)	3.91	3.18–4.81		5.60	4.48–7.01	
Q4 (>28)	18.81	15.5–22.9		8.34	6.62–10.5	
Continuous (Per 5 unit)	1.27	1.25–1.29	P < 0.001	1.28	1.24–1.33	P < 0.001
**ALP**						
Q1 (<66)	referent			referent		
Q2 (66–78)	1.24	1.08–1.41		2.04	1.64–2.54	
Q3 (78–93)	1.59	1.40–1.80		3.14	2.51–3.92	
Q4 (>93)	2.33	2.06–2.63		5.68	4.55–7.13	
Continuous (Per 5 unit)	1.07	1.06–1.08	P < 0.001	1.16	1.13–1.18	P < 0.001

b adjusted for age, and education level.

The robustness of these findings was evaluated by sensitivity analyses. First, Additional adjustments were conducted for the a variety of factors which were shown in Table 1. None materially changed the trend, and none of the risk estimates changed by more than 5% when included. Second, we restrict the liver enzymes to levels of within-normal-limits. The significant trend kept and the risk estimates became stronger (Table 4).

**Table 4:** Logistic regression analysis for the presence of MetS in relation with liver enzymes within-normal-limits

Variables (Per 5 unit)	OR^c^	95% CI	P value
**ALT**	1.58	1.53–1.62	P < 0.001
**AST**	1.43	1.38–1.48	P < 0.001
**GGT**	1.64	1.60–1.69	P < 0.001
**ALP**	1.11	1.09–1.12	P < 0.001

c adjusted for age, gender, and education level

## DISCUSSION

In this large, population-based case-control study with 6,268 MetS subjects and 6,330 healthy controls, we systematically evaluated levels of four liver enzymes, both in overall populations and in subjects with liver enzymes within-normal-limits, with MetS risk using both quartiles and continuous unit of liver enzymes. We also conducted stratified analyses by gender for the relations between MetS risk and liver enzymes. Significant associations were detected for all above analyses, and mutual adjustment proved the independence of the relations for all four liver enzymes. Sensitivity analyses didn’t materially changed the trend. To the best of our knowledge, this study should be the largest, which aimed at evaluating the association between liver enzymes measures and MetS risk, and the findings filled the inadequacies of past data.

MetS can increase the risk of cardiovascular diseases and diabetes [26], and nonalcoholic fatty liver disease (NAFLD) has recently been recognized as one of the leading causes of MetS, diabetes, and cardiovascular diseases [27]. Considering serum biomarkers of liver enzymes being sensitive in the detection of NAFLD, it could be hypothesized that liver enzymes might be novel candidate biomarkers for MetS and its clinical outcomes. GGT and ALT chould be used to predict the deposition of fat in liver cells and, therefore, indicating a change in visceral fat [28]. Changes in visceral fat were achieved through inactivation of PPAR, then followed by MetS, insulin resistance, atherosclerosis and other cardiovascular [29, 30]. Previous epidemiological studies [20, 27, 31–42] have also suggested that liver enzymes showed high sensitivity to metabolic disorders, and liver enzymes as a diagnostic tool for the MetS is also very important, although using small sample size.

In current study, we compares the levels of liver enzymes (ALT, AST, GGT, and ALP) between the healthy population and subjects with MetS, using a relatively large sample size (6,268 MetS subjects and 6,330 healthy controls). ORs values and corresponding confidence intervals (95% CI) by quartile method and continuous unit of liver enzymes. The results confirmed that liver enzyme levels of people with metabolic syndrome were far higher than those of healthy population, which further confirmed the existence of a link between the liver enzymes and MetS risk. At the same time, all of the study population were grouped according to gender. After being corrected for age and educational level, the results have shown that all the associations were statistically significant (*p* < 0.001). No matter in male, or in female population, liver enzyme levels were positively correlated with MetS risk. The results of this study could better improve the historical data to determine the relationship between liver enzymes and MetS development, and provide a more robust evidence to support the clinical diagnosis of the metabolic syndrome. Further, some studies [36, 38, 41] indicated that ALT level within the normal range was associated with the metabolic syndrome and its components. This indicated that in clinical diagnosis, the level of liver enzymes might change without departing from the normal range, but may indicate early metabolic disorder occurs. Thus, in the sensitivity analyses, we also tried to restrict the liver enzymes to levels of within-normal-limits. Then, the significant trend of the relations kept and the risk estimates became stronger, which is consistent with the previous findings.

Strengths of this study included the large sample size, the population-based study design, the high participation rate, the homogeneous ethnic background. Further studies are warranted to confirm our findings, and results derived from other populations are needed to better understand the complicated mechanisms of MetS. In conclusion, this study determined that the liver enzyme levels are indeed associated with the MetS risk, both in overall populations and in subjects with liver enzymes within-normal-limits. Combined with previous findings, the results can better support that liver enzyme levels can be used as clinical predictors of MetS.

## MATERIALS AND METHODS

The methods were carried out in “accordance” with the approved guidelines.

### Definition of metabolic syndrome

The MetS was defined using the modified National Cholesterol Education Program/Adult Treatment Panel III criteria for Asian Americans as having ≥3 of the following components [6]: waist circumference ≥90 cm in men or ≥80 cm in women; triglycerides ≥1.7 mmol/L; HDL cholesterol <1.03 mmol/L in men or <1.30 mmol/L in women; blood pressure ≥130/85 mm Hg or taking antihypertensive medications; or fasting glucose ≥5.6 mmol/L, or taking antidiabetic medications.

### Study population

The present study is part of the Health Survey of Adolescent Population in Chongqing (HSAPC), China, an ongoing project conducted at the first affiliated hospital of Third Military Medical University since March, 2011. This is a relatively large cohort of individuals who visit us for a routine annual check-up. Written informed consent for participation according to the instructions of the institutional ethics committee. Epidemiological survey was conducted using a structured questionnaire. Until July, 2014, a total of 57,141 subjects were included in this cohort. According to the definition of MetS above, 6,268 subjects free of diabetes mellitus and liver diseases were diagnosed as MetS. Healthy controls (without any of the five components of MetS above, *N* = 6,330) were frequency matched with the MetS subjects by residency area, gender, and age group (±5 years old). Fasting peripheral venous EDTA blood samples were collected and centrifuged at 4°C and 3000 rpm for 15 minutes. Human participant Institutional Review Board (IRB) approval was obtained from the IRB of Third Military Medical University.

### Measurements

The waist circumference (WC) was measured at a level midway between the lowest lateral border of the ribs and the uppermost lateral iliac crest in standing position. Blood pressure was measured manually by a calibrated aneroid sphygmomanometer. The mean of all three values were used as the systolic (SBP) and diastolic blood pressure (DBP). Plasma fasting glucose, total cholesterol, high-density lipoprotein (HDL) cholesterol, low-density lipoprotein cholesterol, triglycerides, and liver enzymes were measured enzymatically on an automatic analyzer (Hitachi High Technology Co, Tokyo, Japan).

### Statistical analyses

All data was summarized as mean (standard deviation [SD]) for the continuous variables and as number of patients (expressed as a percentage) in each group for the categorical variables. Characteristics of the study population between MetS subjects and healthy controls were compared using the t test and chi-square test as appropriate. In order to characterize the population, we divided the subjects into quintiles of each of the four liver enzymes according to the distribution among healthy controls. Unconditional logistic regression models were used to evaluate the association between quartiles of liver enzymes and risk of MetS. Adjusted odds ratios (ORs) and their corresponding 95% confidence intervals (CIs) were derived from logistic regression models after adjusting for potential confounders including age, gender, and education level. All statistical tests were 2-sided and 0.05 was set as the cut point of P value. All analyses were conducted using SAS, version 9.3 (SAS Institute, Cary, North Carolina).

## References

[1] Dekker JM, Girman C, Rhodes T, Nijpels G, Stehouwer CD, Bouter LM, Heine RJ (2005). Metabolic syndrome and 10-year cardiovascular disease risk in the Hoorn Study. Circulation.

[2] Zhuo Q, Yang W, Chen J, Wang Y (2012). Metabolic syndrome meets osteoarthritis. Nature reviews Rheumatology.

[3] Manco M (2011). Metabolic syndrome in childhood from impaired carbohydrate metabolism to nonalcoholic fatty liver disease. J Am Coll Nutr.

[4] Ruderman NB, Carling D, Prentki M, Cacicedo JM (2013). AMPK, insulin resistance, and the metabolic syndrome. J Clin Invest.

[5] Ford ES, Giles WH, Dietz WH (2002). Prevalence of the metabolic syndrome among US adults: findings from the third National Health and Nutrition Examination Survey. Jama.

[6] Grundy SM, Cleeman JI, Daniels SR, Donato KA, Eckel RH, Franklin BA, Gordon DJ, Krauss RM, Savage PJ, Smith SC, Spertus JA, Costa F (2005). Diagnosis and management of the metabolic syndrome: an American Heart Association/National Heart, Lung, and Blood Institute Scientific Statement. Circulation.

[7] Huang G, Coviello A (2012). Clinical update on screening, diagnosis and management of metabolic disorders and cardiovascular risk factors associated with polycystic ovary syndrome. Current opinion in endocrinology, diabetes, and obesity.

[8] Miller EL, Mitchell A (2006). Metabolic syndrome: screening, diagnosis, and management. J Midwifery Womens Health.

[9] Kotronen A, Yki-Jarvinen H (2008). Fatty liver: a novel component of the metabolic syndrome. Arterioscler Thromb Vasc Biol.

[10] Goessling W, Massaro JM, Vasan RS, D’Agostino RB, Ellison RC, Fox CS (2008). Aminotransferase levels and 20-year risk of metabolic syndrome, diabetes, and cardiovascular disease. Gastroenterology.

[11] Wannamethee SG, Shaper AG, Lennon L, Whincup PH (2005). Hepatic enzymes, the metabolic syndrome, and the risk of type 2 diabetes in older men. Diabetes Care.

[12] Ioannou GN (2008). Implications of Elevated Serum Alanine Aminotransferase Levels: Think Outside the Liver. Gastroenterology.

[13] Seppala-Lindroos A, Vehkavaara S, Hakkinen AM, Goto T, Westerbacka J, Sovijarvi A, Halavaara J, Yki-Jarvinen H (2002). Fat accumulation in the liver is associated with defects in insulin suppression of glucose production and serum free fatty acids independent of obesity in normal men. J Clin Endocrinol Metab.

[14] Park EY, Lim MK, Oh JK, Cho H, Bae MJ, Yun EH, Kim DI, Shin HR (2013). Independent and supra-additive effects of alcohol consumption, cigarette smoking, and metabolic syndrome on the elevation of serum liver enzyme levels. PLoS One.

[15] Lee K, Yang JH (2013). Which liver enzymes are better indicators of metabolic syndrome in adolescents: the Fifth Korea National Health and Nutrition Examination Survey, 2010. Metab Syndr Relat Disord.

[16] Koskinen J, Magnussen CG, Kahonen M, Loo BM, Marniemi J, Jula A, Saarikoski LA, Huupponen R, Viikari JS, Raitakari OT, Juonala M (2012). Association of liver enzymes with metabolic syndrome and carotid atherosclerosis in young adults. The Cardiovascular Risk in Young Finns Study. Ann Med.

[17] Xia MF, Yan HM, Lin HD, Bian H, Pan BS, Yao XZ, Li RK, Zeng MS, Gao X (2011). Elevation of liver enzymes within the normal limits and metabolic syndrome. Clin Exp Pharmacol Physiol.

[18] Villegas R, Xiang YB, Elasy T, Cai Q, Xu W, Li H, Fazio S, Linton MF, Raiford D, Zheng W, Shu XO (2011). Liver enzymes, type 2 diabetes, and metabolic syndrome in middle-aged, urban Chinese men. Metab Syndr Relat Disord.

[19] Kawada T (2011). Liver enzymes, metabolic syndrome, and insulin resistance. J Diabetes.

[20] Steinvil A, Shapira I, Ben-Bassat OK, Cohen M, Vered Y, Berliner S, Rogowski O (2010). The association of higher levels of within-normal-limits liver enzymes and the prevalence of the metabolic syndrome. Cardiovascular diabetology.

[21] Taki K, Nishio K, Hamajima N, Niwa T (2008). Metabolic syndrome defined by new criteria in Japanese is associated with increased liver enzymes and C-reactive protein. Nagoya J Med Sci.

[22] Perera S, Lohsoonthorn V, Jiamjarasrangsi W, Lertmaharit S, Williams MA (2008). Association Between Elevated Liver Enzymes and Metabolic Syndrome Among Thai Adults. Diabetes & metabolic syndrome.

[23] Forlani G, Di Bonito P, Mannucci E, Capaldo B, Genovese S, Orrasch M, Scaldaferri L, Di Bartolo P, Melandri P, Dei Cas A, Zavaroni I, Marchesini G (2008). Prevalence of elevated liver enzymes in Type 2 diabetes mellitus and its association with the metabolic syndrome. J Endocrinol Invest.

[24] Liu M, Yan HM, Gao X, Gao J (2007). [Association of abnormality of liver enzymes and metabolic syndrome in patients with nonalcoholic fatty liver disease]. Zhonghua Yi Xue Za Zhi.

[25] Nannipieri M, Gonzales C, Baldi S, Posadas R, Williams K, Haffner SM, Stern MP, Ferrannini E (2005). Liver enzymes, the metabolic syndrome, and incident diabetes: the Mexico City diabetes study. Diabetes Care.

[26] Leiter LA, Fitchett DH, Gilbert RE, Gupta M, Mancini GB, McFarlane PA, Ross R, Teoh H, Verma S, Anand S, Camelon K, Chow CM, Cox JL, Despres JP, Genest J, Harris SB (2011). Identification and management of cardiometabolic risk in Canada: a position paper by the cardiometabolic risk working group (executive summary). The Canadian journal of cardiology.

[27] Goessling W, Massaro JM, Vasan RS, D’Agostino RB, Ellison RC, Fox CS (2008). Aminotransferase Levels and 20-Year Risk of Metabolic Syndrome, Diabetes, and Cardiovascular Disease. Gastroenterology.

[28] Kain K, Carter AM, Grant PJ, Scott EM (2008). Alanine aminotransferase is associated with atherothrombotic risk factors in a British South Asian population. Journal of thrombosis and haemostasis : JTH.

[29] Despres JP, Lemieux I, Bergeron J, Pibarot P, Mathieu P, Larose E, Rodes-Cabau J, Bertrand OF, Poirier P (2008). Abdominal obesity and the metabolic syndrome: contribution to global cardiometabolic risk. Arteriosclerosis, thrombosis, and vascular biology.

[30] Tenenbaum A, Fisman EZ, Motro M (2003). Metabolic syndrome and type 2 diabetes mellitus: focus on peroxisome proliferator activated receptors (PPAR). Cardiovascular diabetology.

[31] Lee K, Yang JH (2013). Which Liver Enzymes Are Better Indicators of Metabolic Syndrome in Adolescents: The Fifth Korea National Health and Nutrition Examination Survey, 2010. Metab Syndr Relat D.

[32] Mostafa SA, Khunti K, Morris DH, Webb D, Srinivasan BT, Davies MJ (2012). Can liver enzymes predict progression from prediabetes to type 2 diabetes independent of factors associated with the metabolic syndrome in White Europeans and South Asians?. Diabetologia.

[33] Koskinen J, Magnussen CG, Kahonen M, Loo BM, Marniemi J, Jula A, Saarikoski LA, Huupponen R, Viikari JSA, Raitakari OT, Juonala M (2012). Association of liver enzymes with metabolic syndrome and carotid atherosclerosis in young adults. The Cardiovascular Risk in Young Finns Study. Annals of Medicine.

[34] Kawada T (2011). Liver enzymes, metabolic syndrome, and insulin resistance. Journal of Diabetes.

[35] Villegas R, Xiang YB, Elasy T, Cai QY, Xu WH, Li HL, Fazio S, Linton MF, Raiford D, Zheng W, Shu XO (2011). Liver Enzymes, Type 2 Diabetes, and Metabolic Syndrome in Middle-Aged, Urban Chinese Men. Metab Syndr Relat D.

[36] Xia MF, Yan HM, Lin HD, Bian H, Pan BS, Yao XZ, Li RK, Zeng MS, Gao X (2011). Elevation of liver enzymes within the normal limits and metabolic syndrome. Clinical and Experimental Pharmacology and Physiology.

[37] Zhang YF, Lu X, Hong J, Chao ML, Gu WQ, Wang WQ, Ning GA (2010). Positive correlations of liver enzymes with metabolic syndrome including insulin resistance in newly diagnosed type 2 diabetes mellitus. Endocrine.

[38] Steinvil A, Shapira I, Ben-Bassat OK, Cohen M, Vered Y, Berliner S, Rogowski O (2010). The association of higher levels of within-normal-limits liver enzymes and the prevalence of the metabolic syndrome. Cardiovascular diabetology.

[39] Barfield E, Liu YH, Kessler M, Pawelczak M, David R, Shah B (2009). The Prevalence of Abnormal Liver Enzymes and Metabolic Syndrome in Obese Adolescent Females with Polycystic Ovary Syndrome. Journal of pediatric and adolescent gynecology.

[40] Kim D, Kim YJ, Kim W, Kim BG, Yoon JH, Lee HS (2008). Elevated Liver Enzymes as a Marker of Metabolic Syndrome and Insulin Resistance; a Cross-Sectional Case-Control Study of 16,621 Healthy Subjects. Hepatology.

[41] Wingeyer SDP, de Larranaga GF, Belli SH, Graffigna MN, Fainboim H (2008). The range of normal values of liver enzymes in the era of metabolic syndrome: the need for a redefinition. Eur J Gastroen Hepat.

[42] Nannipieri M, Gonzales C, Baldi S, Posadas R, Williams K, Haffner SM, Stern MP, Ferrannini E (2005). Liver enzymes, the metabolic syndrome, and incident diabetes: The Mexico City Diabetes Study. Diabetes Care.

